# Stathmin levels alter PTPN14 expression and impact neuroblastoma cell migration

**DOI:** 10.1038/s41416-019-0669-1

**Published:** 2019-12-06

**Authors:** Sela T. Po’uha, Marion Le Grand, Miriam B. Brandl, Andrew J. Gifford, Gregory J. Goodall, Yeesim Khew-Goodall, Maria Kavallaris

**Affiliations:** 1 Children’s Cancer Institute, Lowy Cancer Research Centre, UNSW Sydney, Kensignton, NSW 2052 Australia; 2 ARC Centre of Excellence in Convergent Bio-Nano Science and Technology, Australian Centre for Nanomedicine, University of New South Wales, Sydney, NSW 2052 Australia; 3 School of Women’s and Children’s Health, Faculty of Medicine, UNSW Sydney, NSW 2052 Australia; 4 Department of Anatomical Pathology (SEALS), Prince of Wales Hospital, Randwick, NSW 2031 Australia; 5 Centre for Cancer Biology, SA Pathology and University of South Australia, Adelaide, SA Australia; 6 Discipline of Medicine and Dept of Molecular and Biomedical Sciences, The University of Adelaide, Adelaide, SA Australia

**Keywords:** Paediatric cancer, Cell migration

## Abstract

**Background:**

Stathmin mediates cell migration and invasion in vitro, and metastasis in vivo. To investigate stathmin’s role on the metastatic process, we performed integrated mRNA–miRNA expression analysis to identify pathways regulated by stathmin.

**Methods:**

MiRNA and gene arrays followed by miRNA-target-gene integration were performed on stathmin-depleted neuroblastoma cells (Ctrl_shRNA_ vs. Stmn Seq2_shRNA_). The expression of the predicted target PTPN14 was evaluated by RT-qPCR, western blot and immunohistochemistry. Gene-silencing technology was used to assess the role of PTPN14 on proliferation, migration, invasion and signalling pathway.

**Results:**

Stathmin levels modulated the expression of genes and miRNA in neuroblastoma cells, leading to a deregulation of migration and invasion pathways. Consistent with gene array data, PTPN14 mRNA and protein expression were downregulated in stathmin- depleted neuroblastoma cells and xenografts. In two independent neuroblastoma cells, suppression of PTPN14 expression led to an increase in cell migration and invasion. PTPN14 and stathmin expression did not act in a feedback regulatory loop in PTPN14- depleted cells, suggesting a complex interplay of signalling pathways. The effect of PTPN14 on YAP pathway activation was cell-type dependent.

**Conclusions:**

Our findings demonstrate that stathmin levels can regulate PTPN14 expression, which can modulate neuroblastoma cell migration and invasion.

## Background

Neuroblastoma is the most common paediatric solid tumour responsible for 15% of all paediatric oncology deaths.^1^ Widely disseminated, metastatic disease at diagnosis is very common and confers a poor prognosis with a 5-year survival rate of <50%.^2^ There is an urgent need to better understand metastasis mechanism and to identify key regulators of metastasis in high-risk neuroblastoma.

Metastasis is the movement of cancer cells from the primary sites via blood vessels and regrowth at distant sites through a highly selective process consisting of a series of discrete and sequential steps modelled into a metastatic cascade.^3^ Despite the focus on the actin cytoskeleton on cancer metastasis, there is increasing evidence that microtubules and their interacting proteins are also involved in this process.^4^ The microtubule-destabilising protein stathmin is a small cytosolic phosphoprotein that is overexpressed in many cancers including neuroblastoma.^5–8^ We have previously shown that stathmin can mediate neuroblastoma cell migration, invasion and transendothelial migration in both in vitro and in vivo neuroblastoma metastasis models.^9,10^ Our studies have also provided the first evidence that stathmin’s influence on neuroblastoma cell migration is in part mediated by RhoA/ROCK signalling in a microtubule-independent manner.^10^ However, how stathmin influences neuroblastoma metastasis has not yet been comprehensively addressed in neuroblastoma.

Growing evidence has revealed that microRNAs (miRNAs) play a role in multiple pathways in cancer and actively contribute to tumour development and progression.^11^ Specific miRNAs, collectively termed ‘metastamirs', have multiple regulatory functions in different steps of the metastatic cascade.^12^ Recent work in neuroblastoma suggests that the deregulation of miRNAs may play an important role in pathogenesis and chemotherapy resistance.^13^ In this study, we hypothesised that new insights into the role of stathmin in metastatic neuroblastoma can be gained by identifying deregulated miRNAs and associated signalling pathways following the alteration of stathmin expression.

We performed functional mRNA and miRNA expression profiling in stathmin-depleted neuroblastoma cells and identified differentially expressed miRNAs and their target genes. We focused on miR-382 and its predicted target gene PTPN14 and its relevance to neuroblastoma migration and invasion. Our results highlight a new role of PTPN14 in neuroblastoma migration and invasion and may represent a new diagnostic and/or therapeutic target for highly metastatic neuroblastoma.

## Materials and methods

### Cell culture

Neuroblastoma cell lines SK-N-BE(2) (obtained from Dr Sylvain Baruchel, The Hospital for Sick Children, Toronto, ON, Canada) and SH-SY5Y (obtained from the late Dr June Biedler, Fordham University, NY, USA) were maintained as monolayer in Dulbecco’s Modified Eagle Medium (Life Technologies) supplemented with 10% foetal calf serum (FCS) and grown in a humidified incubator at 37 °C and 5% CO_2_. SK-N-BE(2) and SH-SY5Y cells stably expressing GFP-TGL-luciferase (hereafter, designated SK-N-BE(2)/TGL and SH-SY5Y/TGL cells) and either co-expressed control (Ctrl_shRNA_) or stathmin short-hairpin RNA (Stmn Seq2_shRNA_ and Seq3_shRNA_) were also utilised.^9,10^ All cell lines were validated by short tandem repeat profiling (CellBank Australia, Westmead, New South Wales, Australia) and were free of mycoplasma contamination.

### miRNA and gene expression profiling

Total RNA was isolated by using the miRNeasy kit (Qiagen, Victoria, Australia) according to the manufacturer's recommendations. RNA concentration and purity were determined by absorbance using the Nanodrop 1000 (Thermo Fisher Scientific, MA, USA). RNA integrity was verified by means of ribosomal RNAs 18S and 28S on a total RNA Nano chip using the Bioanalyzer (Aligent, CA, USA). Only samples with a RNA integrity number (RIN) greater than 9 were used for array analysis. Labelling and hybridisation to Affymetrix Human 2.0 Gene ST arrays for gene expression profiling, and to Affymetrix Gene Chip miRNA 3.0 arrays (based on miRBase v17) for miRNA expression profiling, was performed by the Ramaciotti Centre for Genomics (University of New South Wales, Sydney, Australia). Three independent samples were prepared from Ctrl_shRNA_ and Stmn Seq2_shRNA_ SK-N-BE(2)/TGL cells and used for hybridisation on separate array chips.

### miRNA and gene expression analysis

Analysis and visualisation of microarray data for genes and miRNAs were conducted using Partek® Genomics Suite TM software (Partek Inc., MO, USA). Background correction, quantile normalisation, log2 transformation and probe set summarisation were performed using default settings for the Robust Multichip Average (RMA) procedure. Quality of chips was assessed using QC metrics. Multidimensional intensity data were explored for differences in samples using principal component analysis (PCA). Differential expression between mRNAs or miRNAs in stathmin-depleted Stmn Seq2_shRNA_ compared with Ctrl_shRNA_ cells was compared by using one-way analysis of variance (ANOVA) with *p*-value < 0.05. Significantly deregulated mRNAs or miRNAs in stathmin-depleted Stmn Seq2_shRNA_ compared with Ctrl_shRNA_ cells were also identified based on the criteria of fold-change FC < −1.4 or FC > 1.4. Multiple comparison correction was performed using a FDR (false discovery rate) of 0.05. Selection of miRNAs and genes, which were differentially expressed in Stmn Seq2_shRNA_ compared with Ctrl_shRNA_, was illustrated using volcano plots. Unsupervised hierarchical clustering was employed for visualisation of patterns in the data. Intensity values were therefore standardised to a mean of zero and scaled to a standard deviation of one. Agglomerative hierarchical clustering of significantly deregulated miRNAs or mRNAs was performed using Euclidean distance to determine row/column dissimilarities, while the distance between two clusters was computed using average linkage. Dendrograms were used to visualise the hierarchy of clusters and identify samples and miRNAs or genes with similar profiles.

### Gene enrichment analysis

Gene set enrichment analysis (GSEA) and Gene Ontology (GO) analysis were performed to identify cellular processes and function in which genes with significantly differential expression are involved in. Enrichment of gene sets and GO analysis was determined based on gene enrichment scores and *p*-values, which indicate if the differentially expressed genes belong to a certain category more often than expected randomly. The GO browser integrated into the Partek software (Partek Inc.) was used to leverage the GO database and inspect the three GO categories: biological process, cellular component and molecular function. The GO enrichment analysis indicated if certain GO terms were overrepresented in the list of differentially expressed genes. Enrichment scores and p-values indicate if the differentially expressed genes belong to a certain category more often than expected randomly. GO terms with enrichment *p*-values < 0.05 were selected as significant.

### Integration of microarray data by correlating differentially expressed miRNAs and target genes

The integration of miRNA and mRNA expression data was performed using a tool integrated in the Partek software (Partek Inc.) and based on miRNA-target-gene databases. Multiple integration analyses were performed with the commonly used target prediction databases Targetscan (version 6.2) (www.TargetScan.org) and Microcosm (version 5) (https://omictools.com/microcosm-targets-tool), and the results were intersected. Only miRNAs and mRNAs, which were previously identified as significant or differentially expressed between Ctrl_shRNA_ and Stmn Seq2_shRNA_, were included in the analysis.

### RNA isolation and real-time quantitative PCR

Total RNA was extracted using RNeasy kit (Qiagen) and reverse transcribed using either the High Capacity cDNA Reverse Transcription kit (ABI, CA, USA) or QuantiTect Reverse Transcription kit (Qiagen) for gene expression analysis. RT-qPCR was performed using a Power SYBR green PCR master mix (Applied Biosystems, CA, USA) with STMN1_1_SG QuantiTect Primer assay (Qiagen), PTPN14 primers (Geneworks) or CYR61_1_SG QuantiTect Primer assay (Qiagen) using an Applied Biosystems 7900HT Fast Real-time PCR System. Stathmin and PTPN14 expression were normalised to that of the housekeeping gene, β2-microglobulin (QuantiTect primer assay, Qiagen).

For miRNA expression analysis, 500 ng of total RNA was reverse transcribed using the Taqman microRNA Reverse Transcription kit (Applied Biosystems) and the Taqman microRNA assays (Applied Biosystems). Preamplification of the reverse transcription product was performed using the Taqman Preamp master mix (Applied Biosystems) according to the manufacturer’s instructions. RT-qPCR was performed using the TaqMan® Universal PCR Master Mix, no AmpErase® UNG (Applied Biosystems) with miR132, miR221, miR222, miR-382, miR-488, miR620, miR-935, miR1281, miR3935, miR-4656, miR4492 and miR4682 Taqman microRNA assays (Applied Biosystems) using an Applied Biosystems 7900HT Fast Real-time PCR System (Applied Biosystems). Expression of miRNA was normalised to the controls RNU48, RNU6B and RNU58A (Taqman microRNA control assays, Applied Biosystems).

### Transfection assays

Target-gene expression was determined in SK-N-BE(2) cells transfected with mirVana miR-382-5p mimic (1 pM) (Ambion, Thermo Fisher Scientific) or mirVana miRNA Mimic Negative control #1 (1 pM) (Ambion, Thermo Fisher Scientific), as well as mirVana miR-382-5p inhibitor (100 nM) (Ambion) or mirVana miRNA Inhibitor Negative control #1 (100 nM) (Ambion) using Lipofectamine 2000 (Life Technologies, Victoria, Australia) according to the manufacturer’s instructions. Cells were harvested 48 h post transfection.

Transfection with siRNAs was performed as previously described.^9^ Briefly, SK-N-BE(2)/TGL and SH-SY5Y/TGL cells were transfected with PTPN14 siRNA sequence 3 (PTPN14 Seq3_siRNA_) (5′-GCUAAUGAGCCUUUGCUUU-3′), sequence 4 (PTPN14 Seq4_siRNA_) (5′-GGUGAGCACUACUCGGAAA-3′) (5 nM) (Dharmacon, CO, USA) or AllStar control siRNA (Qiagen) using Lipofectamine 2000 (Life Technologies) according to the manufacturer’s instructions.

### Western blot analysis

Protein extraction was carried out as previously described.^14^ Fractionation of nuclear and cytoplasmic protein was carried out using the NE-PER^TM^ nuclear and cytoplasmic fraction reagent (Thermo Fisher Scientific) according to the manufacturer’s instructions. Fifteen micrograms of nuclear protein lysates and equivalent amount of the cytoplasmic protein lysates were separated on 4–15% Precast PAGE gels (Bio-Rad) and electrotransferred to nitrocellulose membranes. The membranes were probed with antibodies against PTPN14 (Sigma-Aldrich, NSW, Australia), YAP (clone D8H1X, Cell Signaling, MA, USA), Taz (clone V386, Cell Signaling), stathmin (BD Bioscience, Victoria, Australia), Topoisomerase I (Novus Biologicals, CO, USA) as a control for nuclear fraction and GAPDH (clone 6C5, Abcam, Victoria, Australia) as a control for equal loading. Proteins were detected by ECL Plus (Pierce, Thermo Fisher Scientific) and membranes were either scanned using the Typhoon (GE Healthcare) or exposed to the film. Densitometry analysis was carried out as previously described.^15^

### Immunohistochemistry

Animal experiments were approved by the Animal Ethics Committee, University of New South Wales (ACEC #13/116B). Tumour sections were obtained from control and stathmin-depleted cells xenograft previously obtained in our lab.^10^ Previously generated shRNA-expressing SK-N-BE(2)/TGL cells (control vs. stathmin)^9^ were injected via the lateral tail vein into 6- to 8-week-old male severe combined immunodeficiency (SCID)-Beige mice. Animals were housed with an inverse 12-h day–night cycle with lights on at 8:30 pm in a temperature- (22 ± 1 °C) and humidity- (55 ± 5%) controlled room. All mice were allowed free access to water and a maintenance diet. For in vivo experiments, at least 10 mice per group were shown to have 88.5% power to detect a 50% decrease in tumour growth and/or metastases in mice implanted with stathmin shRNA neuroblastoma cells (*α* = 0.05). Mice were humanely killed, and organs and tumours collected at 23 days post neuroblastoma cell injection or earlier if the mice were of ill thrift. Immunohistochemistry was carried out as previously described.^9^ Tumour sections obtained from control and stathmin-depleted cells xenograft were incubated with PTPN14 antibody or rabbit ImmunoglobulinG control antibody at the same concentration followed by incubation with 3,3′-Diaminobenzidine as a substrate for the peroxidase reaction and haematoxylin as the counterstain. Four individual mouse samples per condition were viewed and imaged using a ×60 objective in an Olympus microscope and analysed using the cellSens software. Ten fields per sample were analysed. Scoring of PTPN14 expression was carried out as previously described.^16^

### Migration and invasion assays

Following PTPN14 or control siRNA 72 h post transfection, migration and invasion assays were carried out as previously described.^9^

### Cell proliferation assay

The PTPN14 or control siRNA-transfected cells were harvested, and total cells were counted by trypan blue exclusion at 24, 48 and 72 h. Analysis of cell growth was carried out as previously described.^17^

### Statistical analysis

Statistical analysis was performed using either two-sided, unpaired Student’s *t* test or one-way ANOVA and values are expressed as mean ± SEM. *p* < 0.05 was considered statistically significant.

## Results

### Differential expression of stathmin modulates both gene and miRNA expression in SK-N-BE(2)/TGL neuroblastoma cells

**Fig. 1:**
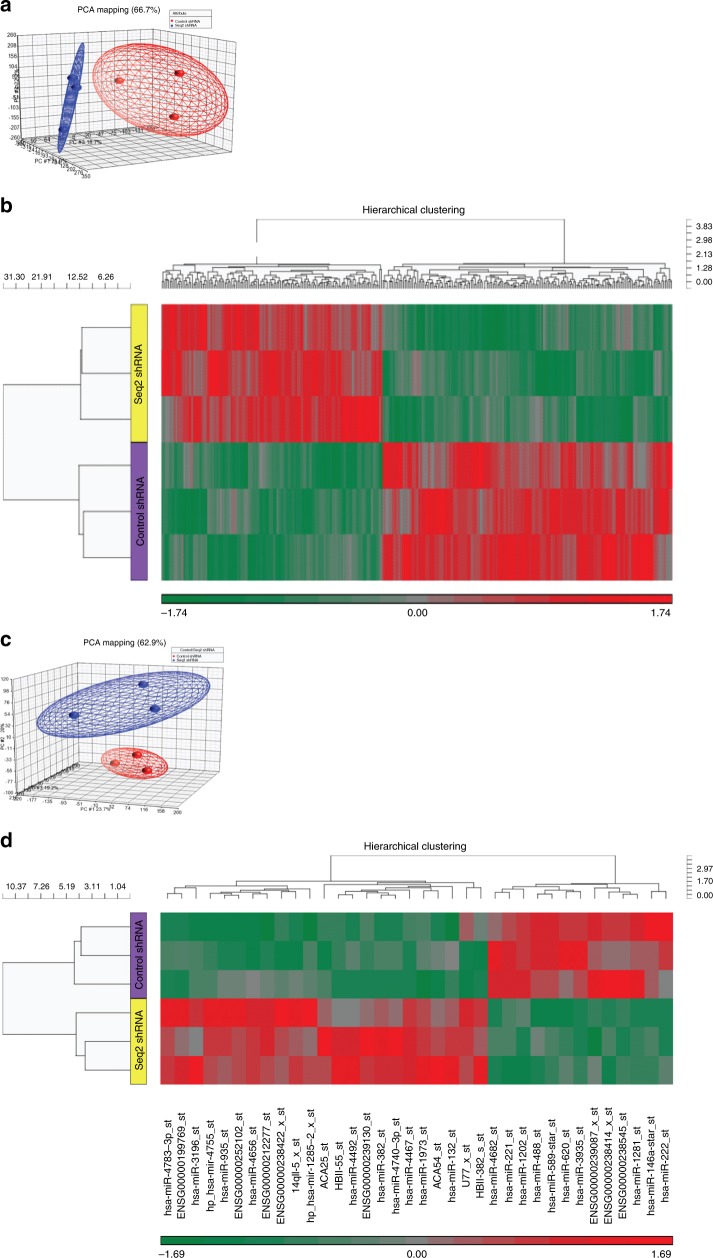
Differential expression of stathmin modulates both gene and miRNA expression in SK-N-BE(2)/TGL neuroblastoma cells. **a** Scatter plot of principal components computed from mRNA expression data exploring high-dimensional data for similarities and dissimilarities between samples. Each of the points in the plot represents a sample (chip). A code colour was used to denote samples derived from CtrlshRNA (red) and Stmn Seq2shRNA (blue). **b** Hierarchical clustering of 324 differentially expressed genes in Stmn Seq2_shRNA_ versus Ctrl_shRNA_ SK-N-BE(2)/TGL cells. Genes are represented in columns and samples are shown in rows. Using average linkage and agglomerative clustering samples and genes with similar profiles were clustered. The resulting dendrogram shows a clear separation of Ctrl_shRNA_ versus Stmn Seq2_shRNA_ samples based on the signature of the 324 differentially expressed genes and allowed for visualisation of genes, which are up-/downregulated in Stmn Seq2_shRNA_ compared with Ctrl_shRNA_ cells. Expression was standardised to a mean of zero and a standard deviation of one. Colours are indicative of gene expression: downregulated genes have negative values and are coloured in green, while upregulated genes have positive values and are coloured in red. **c** Scatter plot of principal components computed from miRNA expression data. Same analysis is used as in **a**. **d** Hierarchical clustering of 36 differentially expressed miRNAs in Stmn Seq2_shRNA_ versus Ctrl_shRNA_ SK-N-BE(2)/TGL cells. Same analysis is used as in **b**.

We have previously shown that stathmin levels are important for the migration and invasion phenotype of neuroblastoma.^9,10^ To further investigate the role of stathmin expression in neuroblastoma metastasis, we used SK-N-BE(2)/TGL neuroblastoma control cells (Ctrl_shRNA_) and their respective stable-knockdown stathmin cells (Stmn Seq2_shRNA_)^9^ (Supplementary Fig. 1a). mRNA and miRNA expression levels in Stmn Seq2_shRNA_ and Ctrl_shRNA_ cells were measured using microarrays. This was followed by the identification of the predicted target genes of these differentially expressed miRNAs (Supplementary Fig. 1b). From microarray analysis, the fold change (FC) in stathmin expression between the Ctrl_shRNA_ and Stmn Seq2_shRNA_ cells was FC = 1.4. Thus, mRNAs were considered differentially expressed between Ctrl_shRNA_ and Stmn Seq2_shRNA_ cells when FC < −1.4 or FC > 1.4, and *p* < 0.05 (ANOVA). To get a first indication of the difference in mRNA expression between the samples, principal component analysis (PCA) was conducted. PCA showed that samples derived from Ctrl_shRNA_ or Stmn Seq2_shRNA_ could be clearly spatially separated into two groups based on their intensity values, indicating differences in gene expression between the samples (Fig. 1a). As visualised using a volcano plot (Supplementary Fig. 2a), 324 genes were identified to be differentially expressed between Stmn Seq2_shRNA_ and Ctrl_shRNA_ cells. This selection of differentially expressed genes included 140 up- and 184 downregulated mRNAs (Supplementary Table 1). To illustrate the difference in mRNA expression, two-way hierarchical clustering was applied to the two sets of data and the 324 deregulated mRNAs. The resulting dendrogram showed that Stmn Seq2_shRNA_ versus Ctrl_shRNA_ samples were clearly separated into two distinct groups based on the mRNA expression profile (Fig. 1b). In the next step, we performed microarray analysis on miRNAs derived from the two cell lines. Like the gene expression data, PCA of miRNA expression (measured as the intensity values derived from miRNA arrays) showed spatial separation between Ctrl_shRNA_ and Stmn Seq2_shRNA_ (Fig. 1c), suggesting that reducing stathmin expression led to alterations in miRNA expression. The criteria for differential expression of miRNAs were as previously defined for mRNA expression (*p*-value < 0.05 and FC < −1.4 or FC > 1.4). Thirty-six miRNAs met these criteria and were therefore considered to be differentially expressed in Stmn Seq2_shRNA_ compared with Ctrl_shRNA_ (Supplementary Fig. 2b). Out of these miRNAs, 23 were upregulated and 13 were downregulated (Supplementary Tables 2, 3). Hierarchical clustering produced a similar dendrogram as observed for the mRNA expression analysis, demonstrating the differences in miRNA profiles between the two sample groups (Fig. 1d).

### Integrated approach to determine miRNAs and their predicted target genes significantly modulated in stathmin-depleted cells

To identify potential targets of miRNAs that are differentially expressed between Stmn Seq2_shRNA_ and Ctrl_shRNA_, we used the miRNA target-gene prediction tools, TargetScan and microCosm. Initially, 13 miRNAs and 40 target genes that were either positively or negatively correlated using Pearson’s correlation were identified. Because miRNAs downregulate expression of their targets, we focused on 12 miRNAs and 20 target genes, which were inversely correlated in their expression. Five of these miRNAs were significantly upregulated, while their target genes were significantly downregulated, and seven miRNAs were significantly downregulated, while reciprocally their target genes were upregulated (Table 1).

**Table 1:** Integration of significantly deregulated miRNAs and target genes with negatively correlated expression.

miRNA	Fold-change Stmn Seq.2shRNA versus CtrlshRNA	*p*-value	Target gene(s)
Downregulated			Upregulated
hsa-miR-3935_st	−2.625	0.0016	GABRA4
hsa-miR-1281_st	−1.7572	0.0056	NMNAT2
hsa-miR-4682_st	−1.6457	0.0078	IBSP. PALM2-AKAP2
hsa-miR-222_st	−1.624	0.0317	NEFH
hsa-miR-221_st	−1.5368	0.0057	INA. NEFH
hsa-miR-620_st	−1.4779	0.0187	VGF. C3AR1
hsa-miR-488_st	−1.4515	0.0099	GPR158. PLEKHH2
**Upregulated**			**Downregulated**
hsa-miR-935_st	1.4656	0.0277	ADAM12. IKZF2. DOCK9. PVRL3. PTPN14
hsa-miR-382_st	1.5146	0.0059	VEGFC. PTPN14
hsa-miR-4656_st	1.7244	0.0173	MARCH3. PTPN14
hsa-miR-132_st	1.8218	0.0405	HUNK. CCDC109B
hsa-miR-4492_st	2.2542	0.0308	STC1

### Modulation of stathmin expression leads to deregulation of migration and invasion pathways

**Fig. 2:**
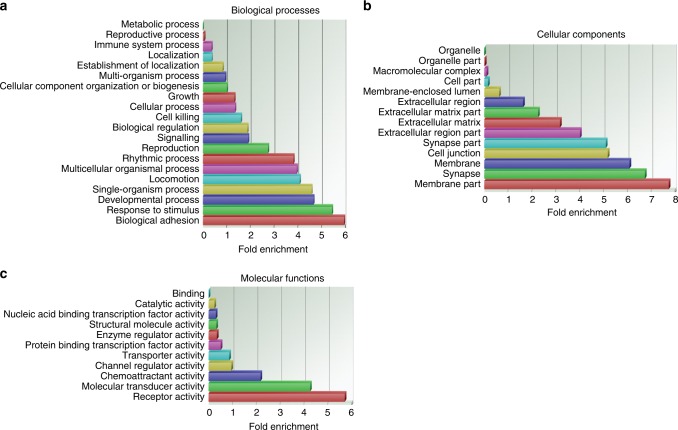
Modulation of stathmin expression leads to deregulation of migration and invasion pathways. Gene ontology (GO) enrichment analysis based on 324 significantly deregulated genes. GO analysis was performed for all three different hierarchical ontologies: **a** biological processes, **b** cellular components and **c**, molecular functions. The enrichment score (*x* axis) was measured if differentially the expressed genes belong to a certain category more often than expected randomly.

To determine the biological and functional significance of the differentially expressed genes, we performed GSEA and GO analysis. GSEA based on the list of 324 deregulated mRNAs revealed eight significant pathways based on KEGG database. Importantly, when the functional analysis was based on only the negatively correlated target genes of miRNAs, the five most deregulated pathways identified included overrepresentation of pathways of ECM-receptor interaction and focal adhesions (Supplementary Table 4). To look closer at functional groupings rather than pathways, GO analysis was conducted taking into account three different hierarchical ontologies (biological processes, cellular components and molecular functions). GO analysis based on all differentially expressed genes indicated that the most enriched biological processes included biological adhesion, response to stimulus and developmental process (Fig. 2a). Focusing on the negatively correlated target genes only, the biological process with the highest enrichment score was developmental process (Supplementary Fig. 3a), including amongst others target genes PTPN14, GABRA4, VEGFC, ADAM12 and PVRL3. The GO category cellular components showed that membrane, membrane part, extracellular region part and cell junction were between the most overrepresented GO terms for both lists of genes (total list of deregulated genes and deregulated negatively correlated target genes, Fig. 2b and Supplementary Fig. 3b). For the GO category, molecular functions, receptor activity and chemoattractant activity were found significantly enriched in both lists of genes (Fig. 2c and Supplementary Fig. 3c). Overall, this functional analysis revealed that the expression profile resulting from the reduction in stathmin expression, entails an enrichment of genes in pathways and GOs that are associated with migration and invasion such as focal adhesion, cell junction and receptor activity.

### miR-382/PTPN14 expression is deregulated in stathmin-depleted cells

**Fig. 3:**
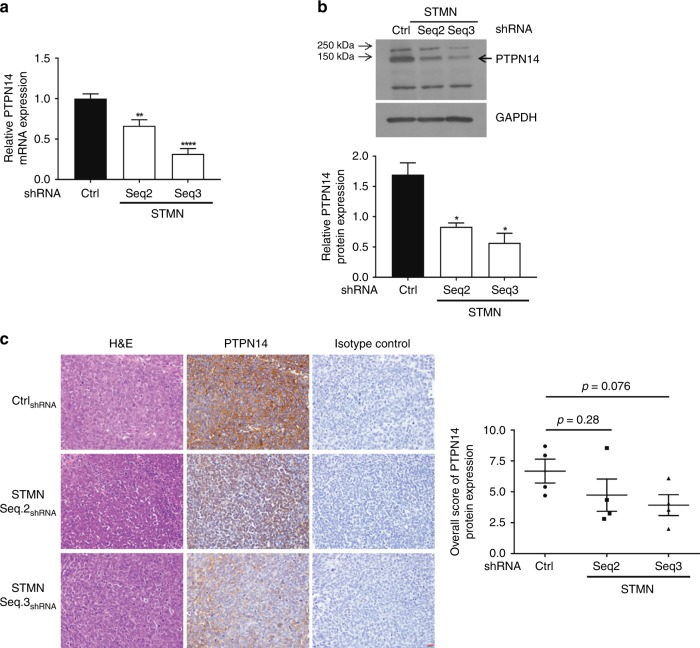
PTPN14 expression is downregulated in stathmin-depleted SK-N-BE(2)/TGL cells and xenografts. **a** RT-qPCR analysis was performed on RNA isolated from the Ctrl_shRNA_, Stmn Seq2_shRNA_ and Stmn Seq3_shRNA_ SK-N-BE(2)/TGL cells. The graph shows the quantitative analysis of PTPN14 gene expression normalised to β2-microglobulin. **b** Representative western blot for PTPN14 protein on whole-cell extracts from the Ctrl_shRNA_, Stmn Seq2_shRNA_ and Stmn Seq3_shRNA_ SK-N-BE(2)/TGL cells. GAPDH was included as a control for equal loading. Graph showing the quantitative analysis of PTPN14 protein expression after normalising to GAPDH. Columns, mean of three independent experiments; bars, SEM. *p < 0.05; **p < 0.01; ****p < 0.0001, statistically significant when comparing the stathmin- depleted cells with the Ctrl_shRNA_. **c** Histology images of tumours from Ctrl_shRNA_, Stmn Seq2_shRNA_ and Stmn Seq3_shRNA_ SK-N-BE(2)/TGL xenografts with haematoxylin and eosin (H&E), PTPN14 isotype control staining. Graph showing the overall scores for PTPN14 protein expression of Ctrl_shRNA_, Stmn Seq2_shRNA_ and Stmn Seq3_shRNA_ SK-N-BE(2)/TGL primary tumours (*n* = 4 individual mouse samples/conditions).

To validate the expression of differentially expressed miRNAs found in the microarrays, qPCR analysis was performed in Ctrl_shRNA,_ Stmn Seq2_shRNA_ and Stmn Seq3_shRNA_ SK-N-BE(2)/TGL cells. Of the 12 differentially expressed miRNAs identified, the trends in differential expression between control and stathmin-depleted cells were validated in three miRNAs (miR-488, miR-382 and miR-4656). MiR-488 expression was downregulated, and expression levels of miR-382 and miR-4656 were upregulated by depletion of stathmin using two different shRNAs (Stmn Seq2_shRNA_ and Stmn Seq3_shRNA_) compared with Ctrl_shRNA_ (Supplementary Table 5). PTPN14 was a predicted target for miR-935, miR-382 and miR-4656, the last two miRNAs showing consistent differential expression profiles by both microarray and qPCR (Table 1 and Supplementary Table 5). Moreover, as miR-382 is predicted by both TargetScan and microCosm to target PTPN14 (Supplementary Fig. 4a), we focused on the functional analysis of miR-382/PTPN14. Consistent with the microarray data, PTPN14 mRNA and protein expression were significantly downregulated in Stmn Seq2_shRNA_ and Stmn Seq3_shRNA_ compared with the Ctrl_shRNA_ cells (Fig. 3a, b). To demonstrate the effect of miR-382 upregulation on PTPN14 expression, we analysed PTPN14 gene and protein expression after transiently transfecting SK-N-BE(2) cells with a miRNA mimic of miR-382. Expression of miR-382 was significantly upregulated with a simultaneous trend to a decrease in PTPN14 gene and protein levels (Supplementary Fig. 4b, c). Conversely, transiently transfecting SK-N-BE(2) cells with a miRNA inhibitor of miR-382 led to decreased expression of miR-382 that was associated with an increase in PTPN14 gene expression (Supplementary Fig. 4d). A trend to downregulate PTPN14 expression was also confirmed in primary tumour samples obtained from stathmin-depleted SK-N-BE(2)/TGL xenografts compared with the Ctrl_shRNA_ tumours (Fig. 3c). Collectively, these results demonstrate that expression of miR-382 and its predicted target PTPTN14 can be modulated by the levels of stathmin in the neuroblastoma cell line SK-N-BE(2).

### Knockdown of PTPN14 expression leads to an increase in neuroblastoma cell migration and invasion

**Fig. 4:**
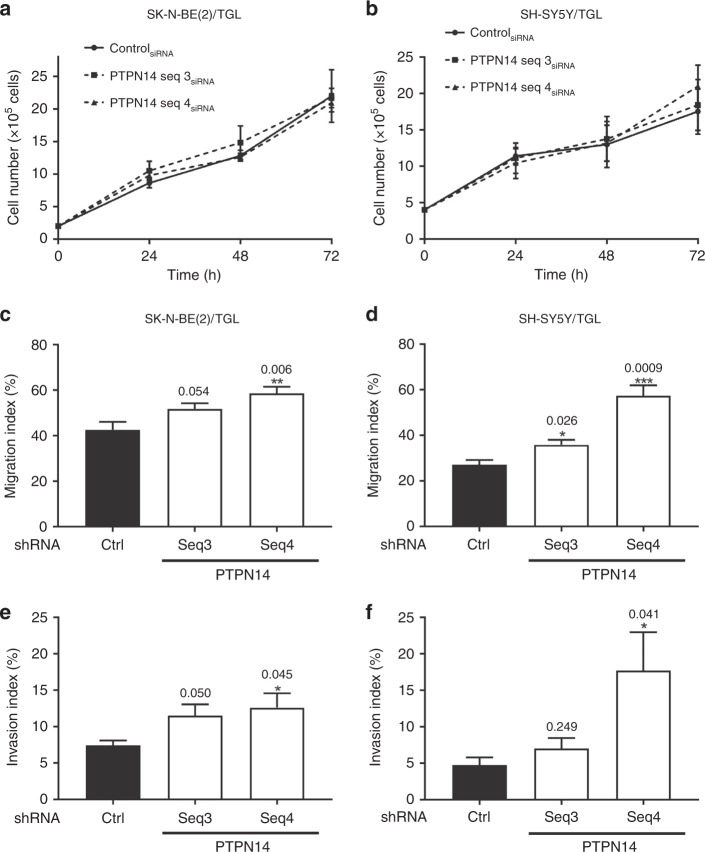
Knockdown of PTPN14 expression leads to an increase in neuroblastoma cell migration and invasion. SK-N-BE(2)/TGL and SH-SY5Y/TGL cells were transfected with two different siRNA sequences targeting PTPN14 (Seq3 and 4). **a**, **b** Proliferation of **a** SK-N-BE(2)/TGL and **b** SH-SY5Y/TGL cells transfected with PTPN14 or control siRNA. **c**, **d** Quantification of migration index in **c**, SK-N-BE(2)/TGL and **d** SH-SY5Y/TGL cells following PTPN14 downregulation 72 h post transfection. **e**, **f** Quantification of invasion index in **e** SK-N-BE(2)/TGL and **f** SH-SY5Y/TGL cells following PTPN14 downregulation 72 h post transfection. Columns, mean of at least three independent experiments; bars, SEM. *p < 0.05; **p < 0.01; ***p < 0.001, statistically significant when comparing the PTPN14-depleted cells with the control.

To understand the role of PTPN14 on cellular functions in neuroblastoma cells, we used a gene-silencing approach in two independent neuroblastoma cell lines. The 72-h time point for PTPN14 treatment resulted in maximal knockdown of the protein (Supplementary Fig. 5a). This time point was used in all subsequent experiments for PTPN14. First, we demonstrated that suppression of PTPN14 did not significantly affect SK-N-BE(2)/TGL and SY-SH5Y/TGL cell proliferation (Fig. 4a, b and Supplementary Fig. 5b–e). Using invasion chambers, our results showed that knockdown of PTPN14 increased the migratory ability of SK-N-BE(2)/TGL cells from 43 ± 8% in control cells to 52 ± 6% (*p* = 0.054) in PTPN14 Seq3_siRNA_ and 59 ± 7% in PTPN14 Seq4_siRNA_ (*p* = 0.006, Fig. 4c) and of SH-SY5Y/TGL cells from 27 ± 4% in control cells to 36 ± 4% (*p* = 0.026) in PTPN14 Seq3_siRNA_ and 57 ± 9% in PTPN14 Seq4_siRNA_ (*p* = 0.0009, Fig. 4d). Moreover, PTPN14 suppression led to a trend to increase in cell invasion of PTPN14-transfected SK-N-BE(2)/TGL and SH-SY5Y/TGL cells compared with the control. Indeed, knockdown of PTPN14 increased the invasion ability of SK-N-BE(2)/TGL cells from 7.4 ± 1% in control cells to 11.5 ± 3% (*p* = 0.05) in PTPN14 Seq3_siRNA_ and 12.6 ± 4% in PTPN14 Seq4_siRNA_ (*p* = 0.045, Fig. 4e) and of SH-SY5Y/TGL cells from 4.7 ± 2.4% in control cells to 7 ± 3.3% (*p* = 0.249) in PTPN14 Seq3_siRNA_ and 17.8 ± 11.7% in PTPN14 Seq4_siRNA_ (*p* = 0.041, Fig. 4f). Altogether, these results show that PTPN14 is involved in neuroblastoma cell migration and invasion without affecting cell proliferation.

### PTPN14 expression regulates the YAP/Hippo signalling pathway in a neuroblastoma cell-type-dependent manner

**Fig. 5:**
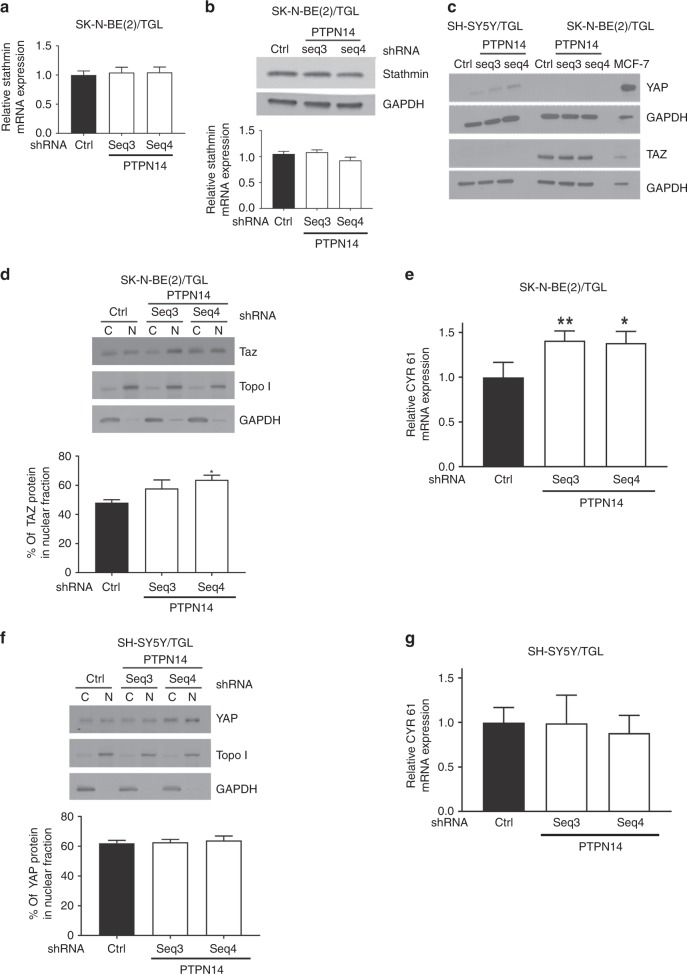
PTPN14 expression regulates the YAP/Hippo signalling pathway in a neuroblastoma cell-type-dependent manner. **a** Relative mRNA expression of stathmin following PTPN14 downregulation in SK-N-BE(2)/TGL cells. Stathmin gene expression normalised to β2-microglobulin. **b** Representative western blot for stathmin protein on whole-cell extracts in SK-N-BE(2)/TGL cells following PTPN14 downregulation. GAPDH was included as a control for equal loading. Graph showing the quantitative analysis of stathmin protein expression after normalising to GAPDH. **c**, Representative western blot showing YAP and Taz expression in the PTPN14-depleted SH-SY5Y/TGL and SK-N-BE(2)/TGL cell lines. GAPDH was included as control for equal loading. The human breast adenocarcinoma cell line MCF-7 was included as a positive YAP/Taz staining. **d** Representative western blot for Taz protein in SK-N-BE(2)/TGL cells following PTPN14 downregulation. GAPDH was included as a control for equal loading in the cellular lysates (**c**) and Topo I for the nuclear fraction (N). Graph showing the quantitative analysis of Taz protein translocated in the nucleus. **e** Relative mRNA expression of CYR61 following PTPN14 downregulation in SK-N-BE(2)/TGL cells. CYR61 gene expression normalised to β2-microglobulin. Columns, mean of at least three independent experiments; bars, SEM. *p < 0.05, **p < 0.01, statistically significant when comparing the PTPN14-depleted cells with the control. **f** Representative western blot for YAP protein in SH-SY5Y/TGL cells as described in **d**. **g** Relative mRNA expression of CYR61 following PTPN14 downregulation in SH-SY5Y/TGL cells as described in **e**.

To further elucidate the signalling pathway involving PTPN14 on neuroblastoma cells, we first investigated the impact of PTPN14 siRNA on stathmin expression. We found no significant alteration in stathmin mRNA and protein expression in the PTPN14- depleted SK-N-BE(2)/TGL (Fig. 5a, b). We then focused on one of the known downstream targets of PTPN14, the Yes-associated protein (YAP or its paralogue Taz), which is a key regulator of the Hippo signalling pathway.^18^ In SH-SY5Y/TGL cells, only YAP was detected, whereas only its paralogure Taz was expressed in SK-N-BE(2)/TGL cells (Fig. 5c). Our results showed that PTPN14 downregulation did not significantly alter the expression of YAP/Taz compared with the control siRNA- transfected cells (Fig. 5c). The activity of YAP/Taz is mediated by its translocation to the nucleus and subsequent transcription of genes that promoted cell proliferation, migration, invasion and metastasis.^19^ We therefore examined the cellular localisation of YAP/Taz in PTPN14-depleted neuroblastoma cells. PTPN14 suppression led to an increase in Taz nuclear translocation, which is significant in PTPN14 Seq4_siRNA_-transfected SK-N-BE(2)/TGL cells compared with the control (Fig. 5d). Analysis of CYR61 mRNA expression, which is one of the target genes of YAP/Taz,^20^ showed a significant increased expression in PTPN14- depleted SK-N-BE(2)/TGL cells (Fig. 5e). However, using another neuroblastoma cell line SH-SY5Y/TGL, our results showed no significant alteration in YAP nuclear translocation and CYR61 mRNA expression in PTPN14-depleted SH-SY5Y/TGL cells compared with the control (Fig. 5f, g). Collectively, these results indicate that PTPN14 and stathmin do not interact in a feed-forward manner and the expression levels of PTPN14 regulate the YAP/Hippo signalling pathway in a neuroblastoma cell line-dependent manner.

## Discussion

Unravelling the mechanisms of metastasis remains a significant biological challenge to neuroblastoma research and highlights the need for mechanistic studies so that ultimately therapeutic vulnerabilities can be targeted. Here, we provide new insight into stathmin’s molecular mechanism in neuroblastoma metastasis. We identified for the first time a role for stathmin in the modulation of genes and miRNAs in neuroblastoma, leading to the enrichment of genes in pathways associated with functions in cell adhesion, migration and invasion. Focusing on one predicted target, we unrevealed a new function of the protein PTPN14 as an inhibitor of neuroblastoma cell migration and identified a potential new target for this metastatic disease (Supplementary Fig. 6).

Stathmin is largely recognised for its ability to interact with tubulin and regulate microtubule dynamics. However, this cytoskeletal protein has also been observed to interact with proteins other than tubulin, many of which have been associated with more migratory and invasive cellular phenotypes such as p27Kip1 and STAT3.^21,22^ Whilst many of the protein functions of stathmin have been reported, its potential role in regulating gene expression and miRNA has been poorly defined. MiRNAs are well known to be a key component driving tumour metastasis in many different cancers including neuroblastoma.^12,13,23,24^ Here, we identified 12 deregulated miRNAs resulting from the stable knockdown of stathmin expression in the SK-N-BE(2) neuroblastoma cells and validated three of them including miR-382, which has been identified as an important regulator of tumour metastasis.^25–28^ We found that miR-382 was upregulated in stathmin-depleted cells that we have previously demonstrated to have a reduced metastatic potential.^9,10^

Our differential and functional gene analysis in stathmin-modulated cells has identified a subset of candidate targets with predicted functions in cell migration and adhesion such as PTPN14,^18^ PVRL3,^29^ ADAM12^30^ and VEGFC.^31^ These results converged into a global modulation of cell–cell and cell–matrix interactions having essential roles in cell motility and migration, confirming that stathmin expression can modulate aggressive, metastatic neuroblastoma in part by regulation of genes with functions in cell migration and adhesion.

The non-receptor tyrosine phosphatase PTPN14 (also known as Pez, PTPD2 and PTP36) is a developmentally regulated non-receptor protein tyrosine phosphatase (PTP).^18^ Its role in cancer has recently emerged as a tumour suppressor. Different studies have shown that PTPN14 modulates cell proliferation and invasion.^32,33^ In addition, a number of its nonfunctional mutations have been reported in breast, pancreas and colorectal tumours.^34–36^ In neuroblastoma, Schramm et al. have found a *PTPN14* nonfunctional mutation in neuroblastoma relapse correlating with an aggressive phenotype.^37^ However, the biological functions of PTPN14 remain poorly characterised. In this study, we identified PTPN14 as one of the predicted targets for three of the differentially expressed miRNAs (miR-382, miR-935 and miR-4656). Our previous studies have shown that downregulation of stathmin led to a decrease in cell migration and invasion in both in vitro and in vivo neuroblastoma metastasis models.^9,10^ Here, we have found that in stathmin-depleted cells, PTPN14 expression is downregulated, suggesting a compensatory mechanism in stathmin-depleted neuroblastoma cells. To determine its independent role in neuroblastoma, we depleted PTPN14 using siRNA in two independent neuroblastoma cell lines. Our results showed an increase in neuroblastoma cell migration and invasion in the absence of any effect on stathmin levels, indicating that PTPN14 and stathmin did not act in a feedback regulatory loop in the PTPN14-depleted cells. These data suggest that downregulation of PTPN14 expression alone enhances cell migration and invasion in neuroblastoma cells. Given our previous findings in stathmin-depleted neuroblastoma cells that showed a significant reduction in cell migration, invasion and metastatic spread,^9,10^ our current results suggest a complex cross talk between signalling pathways involved in the metastatic process. Further studies to understand the interrelationship between stathmin and PTPN14 expression and the migration/invasion phenotype in neuroblastoma are warranted.

Previous work demonstrated that PTPN14 can suppress YAP/Taz activity by promoting its cytoplasmic localisation.^38^ Recently, Cui et al. demonstrated that miR-4516 exerts its oncogenic function by directly targeting PTPN14-mediated regulation of Hippo pathway in glioblastoma.^39^ In addition, Schramm et al. showed that expressing the inactive mutant form of PTPN14 caused nuclear translocation of YAP and enhanced clonogenicity of SK-N-SH neuroblastoma cells.^37^ Our study indicates a neuroblastoma cell-type-dependent molecular consequence of PTPN14 inactivity on YAP/Taz signalling, which might rely on the physiological amount of the YAP/Taz in the nucleus. However, given the modest effect on YAP signalling, our findings also suggest that the effects of PTPN14 knockdown on neuroblastoma migration and invasion could be mediated by an alternative pathway. In support of an alternate pathway, a recent study in breast cancer cells showed that PTPN14 can reduce the secretion of a suite of pro-metastatic factors by altering protein trafficking.^40^

A previous study has reported that PTPN14 regulates cell proliferation in neuroblastoma.^37^ The lack of effect of PTPN14 on cell proliferation in our study may be due to the functionally different methods used for inactivating PTPN14. Our study used RNAi- mediated suppression of PTPN14 expression, whereas Schramm et al. inactivated PTPN14 by mutation.^37^ Mutated proteins can confer distinct functions on cells that differ from depletion of a protein. To date, no chemical compounds have been developed to target stathmin or PTPN14. Understanding the regulatory mechanisms associated with metastasis has the potential to reveal promising therapeutic strategies against stathmin or PTPN14.

## Conclusions

Metastatic neuroblastoma remains a major clinical challenge. Our study provides the first evidence that stathmin can modulate the expression of miRNA and mRNA in neuroblastoma cells, leading to an enrichment of genes involved in migration and invasion signalling pathways. Our data suggest that PTPN14 expression could play a role in the aggressiveness of neuroblastoma and may provide potential new treatment strategies.

## Supplementary information

Supplementary figure legends

Suppl Figure 1

Suppl Figure 2

Suppl Figure 3

Suppl Figure 4

Suppl Figure 5

Suppl Figure 6

Suppl Table 1

Suppl Table 2

Suppl Table 3

Suppl Table 4

Suppl Table 5

## Data Availability

The data that support the findings of this study are available on request from the authors.
