# Supplementary material for: Stathmin levels alter PTPN14 expression and impact neuroblastoma cell migration
Source: Br J Cancer. 2019 Dec 6;122(3):434–44. doi: 10.1038/s41416-019-0669-1 (PMC7000740; doi:10.1038/s41416-019-0669-1)
Supplement: Supplementary file 1 — Supplementary figure legends [file 41416_2019_669_MOESM1_ESM.doc]

**SUPPLEMENTARY FIGURE LEGENDS**

**Supplementary figure 1: a,** Stathmin gene expression in control Ctrl_shRNA_ and Stmn Seq2_shRNA_ SK-N-BE(2)/TGL cells was measured by RT-qPCR. Columns, mean of three independent experiments; bars, SEM. ****p<0.0001. **b,** Flow diagram showing the experimental plan for mRNA and miRNA microarray based analysis followed by a miRNA-target gene-integration in SK-N-BE(2)/TGL neuroblastoma cells with stable stathmin suppression and their respective control cells.

**Supplementary figure 2: a, b** Volcano plot illustrating the magnitude of expression change (fold-change on x-axes) *versus* its statistical significance (p-value on y-axis) for each a, mRNA and b, miRNA. Each point in the plot represents a mRNA or miRNA. mRNAs and miRNAs illustrated as triangles met the cut-off criteria of FC<-1.4 or FC>1.4 and p<0.05.

**Supplementary figure 3:** Gene ontology (GO) enrichment analysis based on the negatively-correlated target genes. GO analysis was performed for **a,** biological processes, **b,** cellular components and **c,** molecular functions. The enrichment score (x-axis) measured if differentially the expressed genes belong into a certain category more often than expected randomly.

**Supplementary figure 4: a,** Predicted alignment by Microcosm of miR-382 with PTPN14 mRNA. **b,** Relative mRNA expression of miR-382 and PTPN14 following 48h miR-382 mimic transfection in SK-N-BE(2)/TGL (n=3; bars, SEM; ****p<0.0001). **c,** Representative Western blot for PTPN14 protein in SK-N-BE(2)/TGL cells following 48h miR-382 mimic transfection. Graph showing the quantitative analysis of PTPN14 protein expression (n=3; bars, SEM). **d,** Relative mRNA expression of miR-382 and PTPN14 following 48h miR-382 inhibitor transfection in SK-N-BE(2)/TGL (n=2; bars, SEM).

**Supplementary figure 5: a,** Representative Western blot for PTPN14 protein on whole-cell extracts in SK-N-BE(2)/TGL following PTPN14 downregulation 48h, 72h or 120h post-transfection. Graph showing the quantitative analysis of PTPN14 protein expression. **b, c** Relative PTPN14 mRNA expression in **b,** SK-N-BE(2)/TGL and **c,** SH-SY5Y/TGL following PTPN14 downregulation 72h post-transfection. **d, e** Representative Western blot for PTPN14 protein on whole-cell extracts in **d,** SK-N-BE(2)/TGL and **e,** SH-SY5Y/TGL following PTPN14 downregulation 72h post-transfection. Graph showing the quantitative analysis of PTPN14 protein expression. Columns, mean of three independent experiments; bars, SEM. *p<0.05; **p<0.01; ***p<0.001.

**Supplementary figure 6:** Flow diagram showing the study design.
