# Supplementary figures and images for: Stathmin levels alter PTPN14 expression and impact neuroblastoma cell migration
Source: Br J Cancer. 2019 Dec 6;122(3):434–44. doi: 10.1038/s41416-019-0669-1 (PMC7000740; doi:10.1038/s41416-019-0669-1)

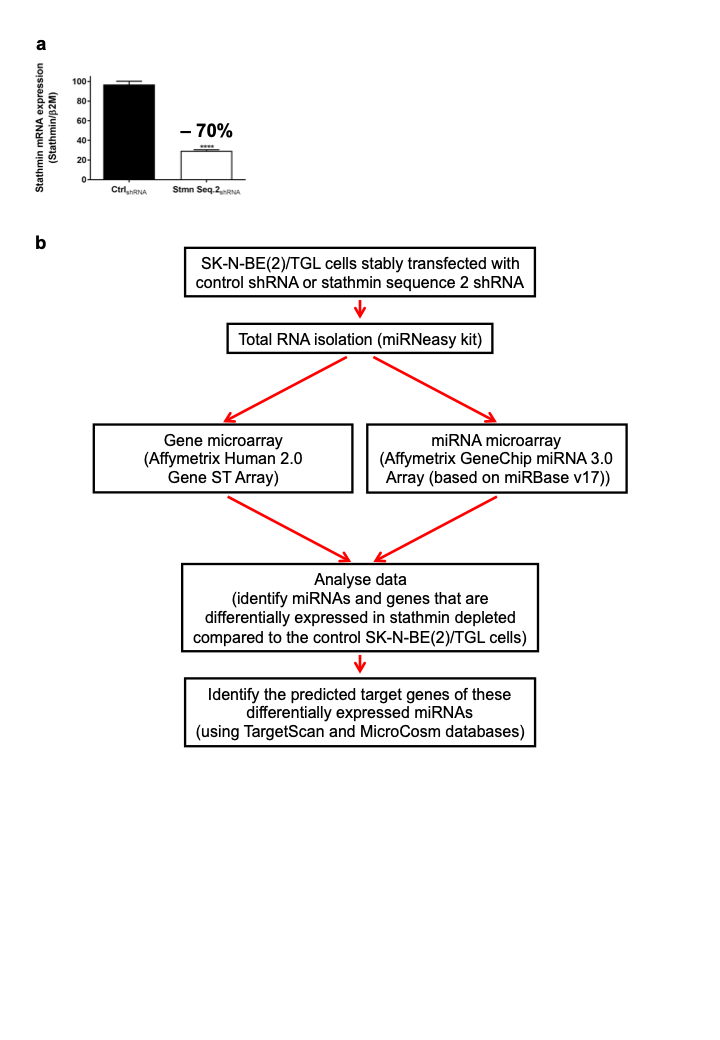

Supplement: Supplementary file 2 — Suppl Figure 1 [file 41416_2019_669_MOESM2_ESM.tif]

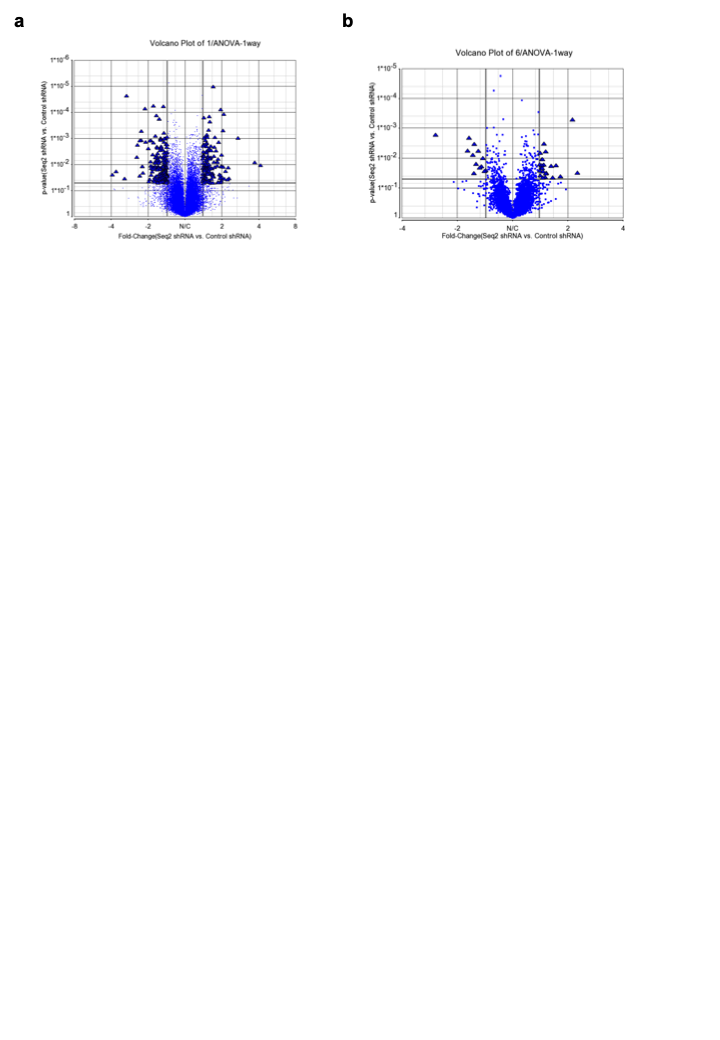

Supplement: Supplementary file 3 — Suppl Figure 2 [file 41416_2019_669_MOESM3_ESM.tif]

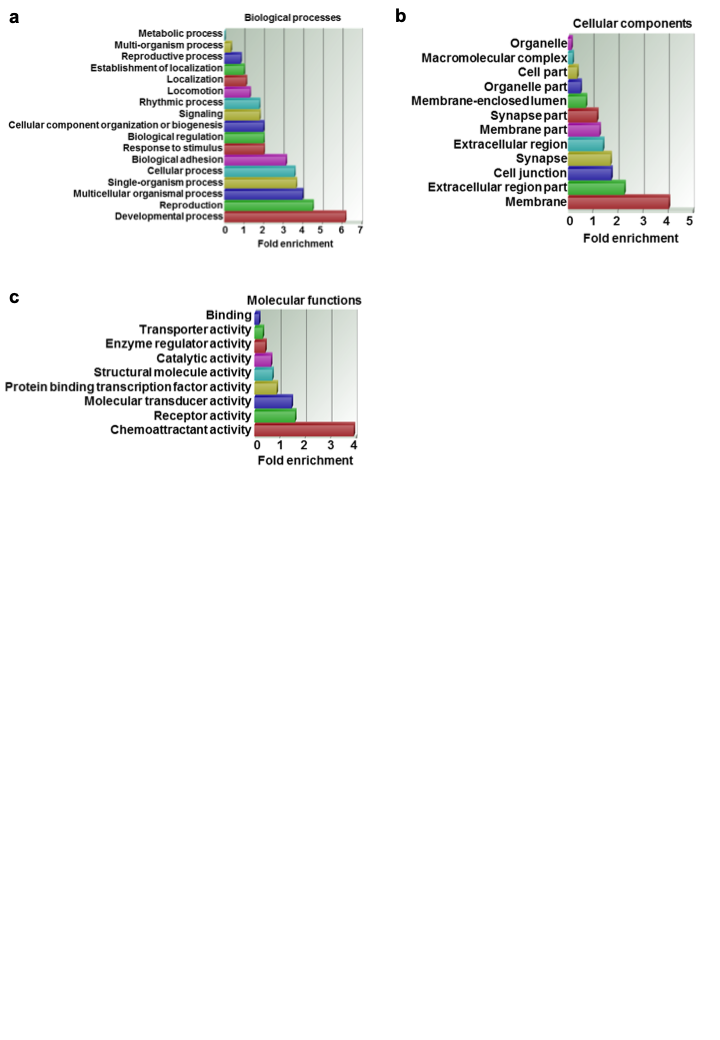

Supplement: Supplementary file 4 — Suppl Figure 3 [file 41416_2019_669_MOESM4_ESM.tif]

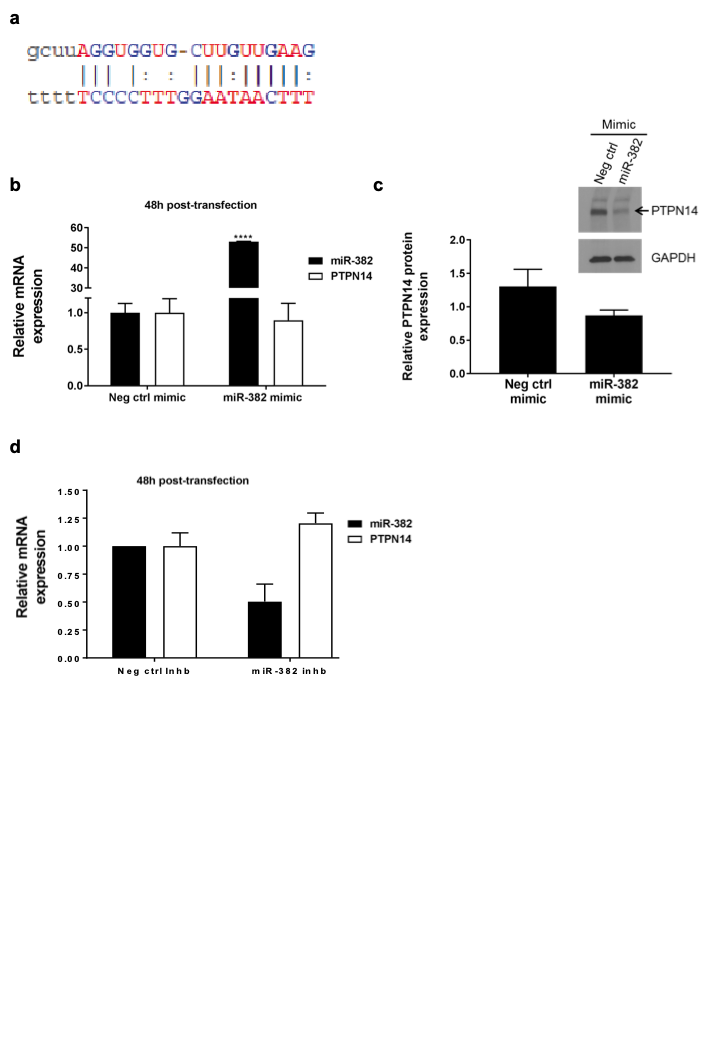

Supplement: Supplementary file 5 — Suppl Figure 4 [file 41416_2019_669_MOESM5_ESM.tif]

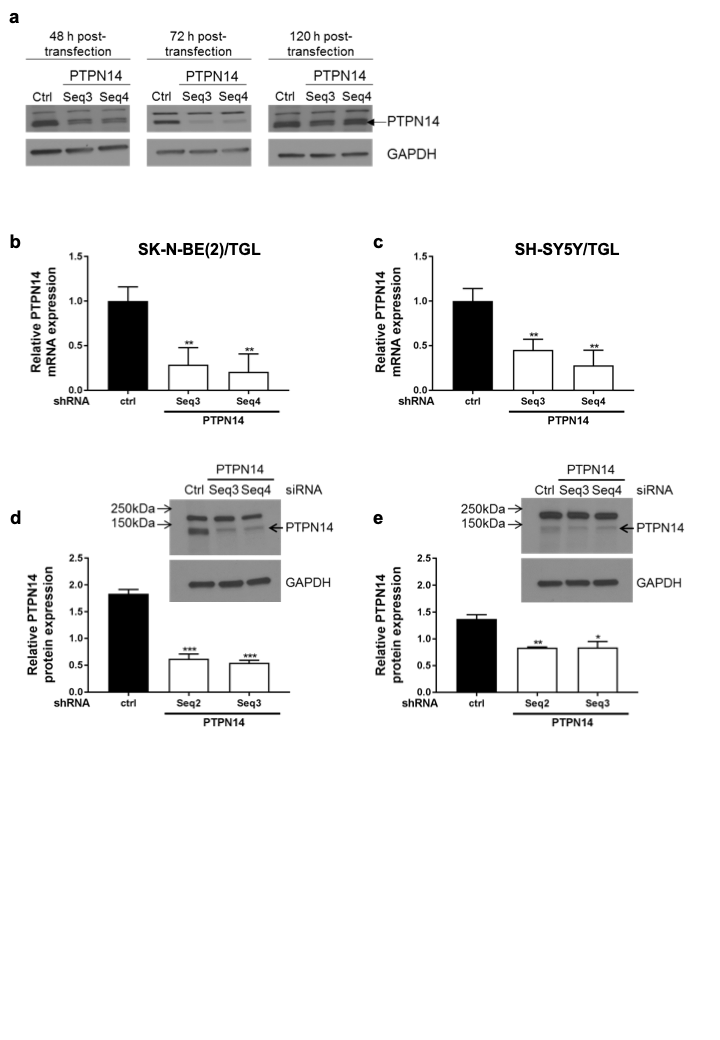

Supplement: Supplementary file 6 — Suppl Figure 5 [file 41416_2019_669_MOESM6_ESM.tif]

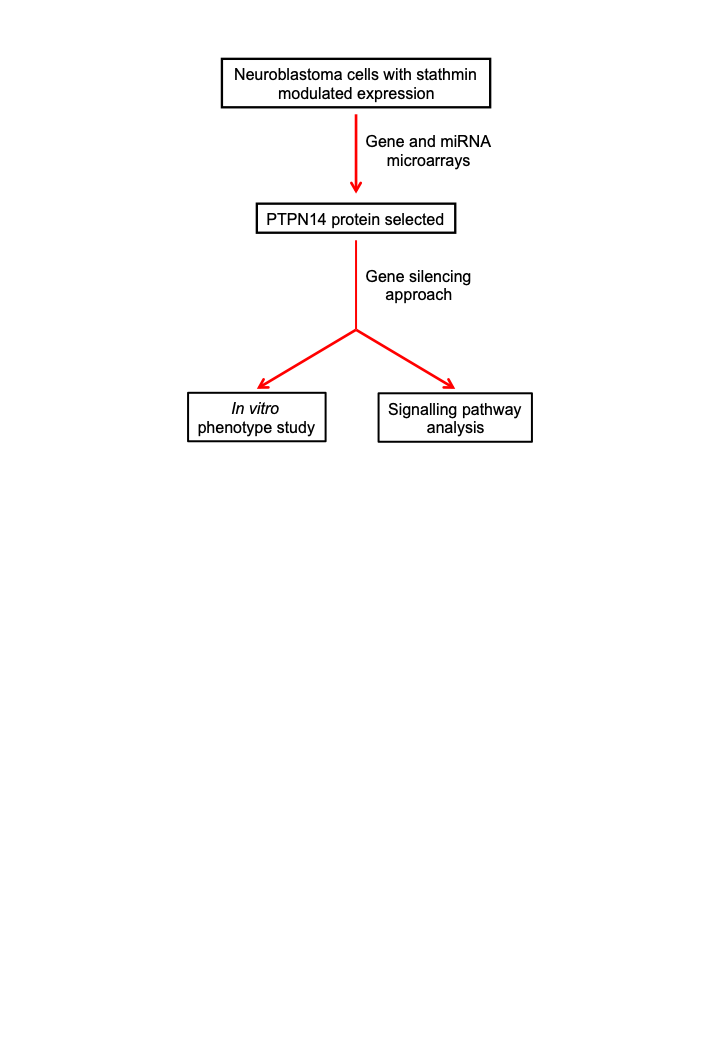

Supplement: Supplementary file 7 — Suppl Figure 6 [file 41416_2019_669_MOESM7_ESM.tif]
